# Beliefs About Return to Work Among Women During/After Long-Term Sick Leave for Common Mental Disorders: A Qualitative Study Based on the Theory of Planned Behaviour

**DOI:** 10.1007/s10926-020-09946-3

**Published:** 2021-01-25

**Authors:** Åsa Hedlund, Eva Boman, Marja-Leena Kristofferzon, Annika Nilsson

**Affiliations:** 1 Department of Caring Sciences, University of Gävle, Kungsbäcksvägen 47, 801 76 Gävle, Sweden; 2 Department of Occupational Health and Psychology, University of Gävle, Kungsbäcksvägen 47, 801 76 Gävle, Sweden

**Keywords:** Common mental disorders, Return to work, Sick leave, Women

## Abstract

**Purpose:**

Long-term sick leave due to common mental disorders (CMDs) is an increasing problem, especially among women. To help these women return to work (RTW) sustainably, we need to know more about their own beliefs about RTW. One applicable theory is the theory of planned behaviour (TPB). Thus, the present study aimed to describe, based on the TPB, women's beliefs about RTW during or after long-term sick leave for a CMD.

**Methods:**

A qualitative approach was used. Twenty women were included during a long-term sick leave period due to a CMD. A deductive content analysis was conducted using predetermined factors from the TPB: behavioural beliefs (advantages–disadvantages of RTW), normative beliefs (supporters and non-supporters of RTW), and control beliefs (facilitators of—barriers to RTW).

**Results:**

The women believed that RTW would give them meaning and balance in life, but also that it would be challenging to maintain balance after RTW. They believed they had several supporters of RTW, but that the support was sometimes perceived as stressful rather than encouraging. Furthermore, individual adaptation and high demands were the most mentioned facilitator and barrier, respectively. Workplace conditions and personal strategies were thought to be important aspects.

**Conclusions:**

By using the TPB, the present study was able to offer new findings on women’s beliefs about RTW after long-term sick leave for a CMD. Based on the findings, we suggest that various RTW stakeholders should focus on striving to provide the tasks and work pace women need so they can maintain their professional competence and sense of meaning.

## Introduction

During recent years, common mental disorders (CMDs), such as depression, stress-related disorders and anxiety, have been a leading cause of disability in many countries, especially among women [1]. Besides the individual suffering and loss of valuable competence on the labour market, the economic cost to society is high [1, 2]. In Sweden today, CMDs cause almost half of the instances of sick leave among women [3], and long-term sick leave due to CMDs has increased for women during the past decade [2]. Studies conducted on women with CMDs have shown that long-term sick leave (> 2 months) is experienced as a painful lesson through which the women discover their “true self”. However, it also harms self-image, self-esteem, and may contribute to feelings of shame [4] and social isolation [5]. Based on this, it is important to identify aspects that enable women to RTW in a sustainable way after long-term sick leave due to a CMD. As a first step, in the present study, we have chosen to study the perspective of the women.

In Sweden, the social insurance agency (SIA), the employer and the health care system, together with the employee, have the responsibility for helping the individual return to work (RTW) after sick leave [2]. However, the SIA has the financial responsibility for individuals during a sick leave period, including regularly assessing whether they can partially and/or fully work or are still entitled to partially and/or fully receive sick leave benefits [6]. The SIA’s assessments are based on a physician’s certificate, including the individual’s medical diagnosis and working capacity [7].

During or after long-term sick leave for a CMD, individuals cope with RTW in different ways. One meta-synthesis showed that one important strategy was to not exceed the limits of one’s working capacity; this was, however, very difficult to accomplish, especially for individuals who had a perfectionist personality or who did not want to be a burden on others at work [8]. Research conducted on women with a CMD showed that they joined self-help groups on the Internet or read self-help books, aiming to better understand their personality. They also reconsidered their life goals, and new values emerged. For example, family life became more important than before [5].

Although individual adaptation plays a role in RTW [9], general workplace factors are also essential. For example, a small workplace, bullying and low decision latitude are negatively associated with RTW, while variety in work and supportive supervisory behaviour are positively associated with it [10]. Furthermore, involvement on the part of the workplace [2, 8], the individual’s contact with the workplace during sick leave and multicomponent interventions such as organizational changes, therapy and a graded RTW can all facilitate RTW among persons with a CMD after sick leave [11]. However, a systematic review among people with various health problems showed that individuals’ own beliefs about RTW also have a significant impact on RTW [12]. For example, fear-avoidance beliefs and catastrophizing were negatively associated with RTW. However, beliefs about RTW have rarely been investigated among persons with a CMD, especially among women on long-term sick leave, who are the most vulnerable group in this context [3]. One study found that individual adaptation to working tasks, the workplace and working hours are perceived as important to RTW [13]. Another study revealed that, among these women, perceived barriers to RTW included conflicts with or lack of support from one’s employer or colleagues, traumatic events in private life, and that their disorder’s invisibility would cause work colleagues to forget their limitations. Reasons for RTW could be maintaining their work-related identity and improving their financial situation, but also worries about being replaced and, therefore, losing their job [14].

Previous studies have called for theory-driven research in this area [15, 16]. An appropriate theoretical framework to use for understanding individuals’ beliefs regarding a specific behaviour, in the present study RTW, is the Theory of Planned Behaviour (TPB) [17]. According to the TPB, the performance of a certain behaviour depends on an individual’s intention to perform the behaviour. This intention, in turn, is determined by motivational factors: behavioural beliefs, normative beliefs, and control beliefs. Behavioural beliefs concern the person’s attitude towards the behaviour, i.e., the advantages and disadvantages believed to be associated with the behaviour and their perceived importance. Normative beliefs concern social pressure, i.e. beliefs about which people in an individual’s life support or do not support the individual performing the behaviour and how important these people’s opinions are perceived to be. Control beliefs concern the individual’s perceived control over the behaviour, i.e., the facilitators or barriers believed to affect performance of the behaviour [17]. However, most behaviours require more than a strong intention, i.e., others’ collaboration, time, and skills [17] or, in the present case, a job to return to.

The TPB has been used for decades to understand a variety of behaviours, often health behaviours such as diet patterns or alcohol consumption [18, 19]. Only a few studies have used the TPB to address RTW, and these studies have focused on persons with musculoskeletal disorders or on sick leave in general [20, 21]. Nevertheless, this indicates that use of the TPB is promising for understanding factors of importance to RTW. An improved understanding of women’s beliefs about RTW after long-term sick leave for a CMD is important if stakeholders are to support RTW among these women. As a first step in this investigation, a qualitative evaluation of these different beliefs is suggested [22]. Therefore, the present study aimed to describe, based on the TPB, women's beliefs about RTW during or after long-term sick leave for a CMD.

## Method

### Design

A descriptive study was conducted, using a qualitative and deductive approach [23, 24].

### Sample and Settings

Consecutive sampling was used, and the inclusion criteria were: women > 18 years; on sick leave full- or part-time for at least the past two months due to a CMD (ICD-codes: F30-F48); able to understand, read and write in Swedish. The exclusion criteria were: severe mental illness such as schizophrenia or other psychotic disorders; unemployed or on sick leave for > 2 years. One hundred and fifty women were invited to participate in the study. Of these, 20 agreed to participate. At the time of the interviews, six were no longer on sick leave.

### Data Collection

Information letters were prepared by the authors and sent to the women by the SIA, which had access to their names and addresses. Two reminders were sent out. The women who wished to participate in the study filled out the invitation form and sent it to the authors. These women were then contacted by the first author, to receive more information about the study; they then had the opportunity to ask questions and decide on a time and location for the interview.

Data were collected using an interview guide with 10 open-ended questions based on factors in the TPB (Table 1). The interview guide was developed by the authors and based on the recommendations proposed by Francis et al. [22]. The first nine questions were structured into three blocks following the TPB factors: behavioural, normative and control beliefs. The tenth question was general and included to complement the previous replies. The interview questions were slightly adapted depending on the women’s situation; i.e., if the women were off work, they were asked about RTW, and if they were working to some extent, they were asked about sustaining work. The first author posed the questions orally, and the women answered orally. Following the recommendations of Francis et al. [22], the women were also asked to write down their answers, briefly, under each question. The written information was used to develop a questionnaire, which will be used in another study. Demographic data, including age, diagnosis and duration of sick leave, were also collected (Table 2). The interviews were conducted in central Sweden from January to May 2018. The interviews were carried out in the individual’s home or in a reserved room at the university; they were audio-recorded and lasted from 34 to 84 (mean 59) minutes.

**Table 1:** The interview guide

The interview questions
1. What do you believe are the advantages of returning to work^a^?
2. What do you believe are the disadvantages of returning to work^a^?
3. Is there anything else you associate with returning to work^a^?
4. Are there any individuals or groups who would approve of you returning to work^a^?
5. Are there any individuals or groups who would disapprove of you returning to work^a^?
6. Concerning individuals or groups, is there anything else you associate with returning to work^a^?
7. What factors or circumstances enable you to return to work^a^?
8. What factors or circumstances make it difficult or impossible for you to return to work^a^?
9. Are there any other issues that come to mind when you think about returning to work^a^?
10. Is there anything else you associate with returning to work^a^?

^a^If the women worked to some extent, “return(ing) to work” was replaced with “remain(ing) in work”

**Table 2:** Participant characteristics (self-reported), *N* = 20

Variables	Numbers
Civil state	
Living with a partner	15
Single	5
Number of children	
No children	3
One child	4
Two children	11
Four children	2
Diagnosis	
Stress-related disorders	13
Anxiety disorders	3
Other^a^	4
Degree of sick leave (%)	
100	4
75–25	10
0	6^b^
Profession	
Health and social care	9
Administrative work	7
School	3
Industrial worker	1
Cause of sick leave *(several women reported more than one cause)*	
Poor work environment	14
Traumatic events in private life	9
High demands on oneself	4
Other^c^	4
History of CMD	
Have been on sick leave for CMD before	11
Have had CMD before but not been on sick leave	3
Have no history of CMD	6

^a^Depression, crisis reaction or feelings of general “mental imbalance”

^b^Three worked full time and three participated in work-orientated rehabilitation or combined work with parental leave/leave of absence

^c^E.g., being a single parent or having difficulties balancing between work and private life

### Data Analysis

The interviews were transcribed verbatim, excluding the identifying information. A deductive content analysis [24] was conducted. In the first step of the analysis, a categorization matrix with the pre-determined factors from the TPB was prepared. After the matrix was developed, data were reviewed for units of analysis that were relevant to the factors in the matrix. The units of analysis were then sorted under each factor in separate documents (behavioural beliefs in one document, normative beliefs in another, and control beliefs in still another). Subsequently, the units of analysis were condensed and labelled with codes. Based on their differences and similarities, the codes were aggregated and sorted into the matrix. The first author was responsible for the analysis, but to strengthen credibility, all four authors read the transcribed interviews and discussed the data several times during the analysis process. No beliefs that did not fit into the matrix emerged from the data.

## Findings

### Participants

The included women (*n* = 20) were between 28 and 63 years of age (median 45). At the time of the interviews, 14 of the women reported still being on sick leave to some extent. Three of the women were working full time, while 3 were participating in work-orientated rehabilitation or combining work with parental leave/leave of absence. The most common diagnosis was stress-related disorders. A majority of the women lived with a partner and had two children. The most common professions among the women were in health and social care. A poor working environment was a commonly reported cause of the sick leave. Most women had suffered from CMDs earlier in life. For demographic data, see Table 2.

An overview of the factors and codes is shown in Table 3. A unique number representing each woman and quotations are inserted into the text to strengthen the credibility of the findings. These quotations are presented with quotation marks and italics.

**Table 3:** Overview of the pre-determined factors (behavioural beliefs including advantages–disadvantages; normative beliefs including supporters and non-supporters; control beliefs including facilitators and barriers) and the codes

Factors	Behavioural beliefs	Normative beliefs	Control beliefs
Codes	*Advantages of RTW*^a^	*Supporters of RTW*	*Facilitators of RTW*
	Belong to a social context	Social insurance agency	Individual adaptation
	Safety	Employer^b^	Strategies
	Self-esteem and competence	Physicians	Positive treatment on the part of others
	Meaning	Society in general	Relief of symptoms
	Balance	Colleagues^c^	Support from family or close friends
		Family and close friends^d^	
		Clients/care recipients/patients	
Codes	*Disadvantages of RTW*	*Non-supporters of RTW*	*Barriers to RTW*
	Difficulties maintaining balance	Employer	High demands
	Feeling insufficient and fear of losing competence	Colleagues	Financial stress
	Energy-consuming to be with colleagues	One’s own children	Symptoms
		Counsellor	Negative treatment on the part of others
			Shame and guilt
			Age
			Unhealthy working environment

^a^Return to work

^b,c,d^Occur in two places within the factor “normative beliefs” because the women believed that these persons/groups could be both supporters and non-supporters of RTW. This is also shown in the text in the results section

### Behavioural Beliefs

Behavioural beliefs concern women’s believed advantages and disadvantages with RTW.

#### Advantages of RTW

The women believed that one of the greatest advantages of RTW was belonging to a social context. This could concern hearing what was said around the coffee table, being invited to a Christmas party or just socializing and having fun with colleagues. This made the women feel healthier. Furthermore, having a salary and fixed routines in daily life were believed to be obvious advantages, as they impart a feeling of safety, both financially and emotionally. This meant, among other things, not having to worry about their current or future personal economy and having a plan to follow in daily life: “It’s really important, set the alarm, get up in the morning, leave and come home. I think it gives some security (19)”. Others believed the advantages were increased self-esteem and competence, for example in the form of feeling needed, making progress in one’s profession and having an identity: “Knowing I’m part of society, that I am somebody, actually (15)”. Working was also believed to give life meaning. This could concern feeling one was part of something bigger than one’s own existence, having a purpose outside the family and doing something valuable for others. Being a role model for one’s children was also believed to be an advantage: “The fact that he sees I’m part of society—that I like to work. Just like his father (6)”. Furthermore, the women believed that working gave physical and psychological balance to life, because it improved their circadian rhythm, regarding sleep and physical activity, and meant getting outside the home regularly, which made them appreciate their home and private life more.

#### Disadvantages of RTW

A majority of the women believed that one disadvantage was the difficulty in maintaining a balance at work or between work and private life after RTW. This could involve being driven and performance-orientated as a person, but unable to maintain that level for more than short periods of the day, without getting very tired. For the women to be able to work at all, their social life, physical activity and/or being with their children had to be given low priority: “I have to compensate with babysitters, so I can’t manage my private life fully” (2). Another perceived disadvantage was feeling insufficient at work and fearing loss of competence. This could concern not feeling they performed well enough in their own or their colleagues’ eyes, or fearing their competence would no longer be utilized due to their limitations, resulting in loss of competence: “There’s this idea, you know, about ending up in a workplace with very low demands. More like an assembly line. That I wouldn’t be able to work with the knowledge I have, but maybe end up at the wrong place” (11). Some women believed being with colleagues could be energy-consuming, especially when colleagues did not understand their condition and therefore expected more of them than they were able to perform. Another side of this was no longer having patience with one’s colleagues: “What I think is most difficult is this restlessness—in the staff room sometimes, when they talk about completely uninteresting things—somehow I can’t deal with it, I almost have a fit” (19).

### Normative Beliefs

Normative beliefs concern persons the women believe support or do not support their RTW.

#### Supporters of RTW

Overall, support from others was two-sided. On one hand, support was believed to be positive and based on what is best for the women. On the other hand, support was believed to be more negative, causing the woman to feel pressured to RTW for the sake of others. Women believed that stakeholders, i.e. the SIA, the employer or the health care system, wanted them to work, but thought this could be expressed in various ways. One woman described how the SIA let her progress at her own pace: “I – have had a case manager who said, ‘sure, but you shouldn’t feel you’ve failed if you aren’t able to advance, instead you can take two steps back’” (2). Other women believed the SIA wanted them to work because their sick leave was too expensive for society, caused more work for SIA staff and worsened sick leave rates. Regarding the women’s employers, some women believed their employer wanted them to work because they were appreciated at work for a special competence they possessed. However, some women believed their employer wanted them back because that meant fewer problems with staffing and administration. The women’s physicians were also believed to be supporters of RTW, primarily because they cared about the women’s well-being and health, but also because they wanted fewer sick-leave patients and relief from the frustrating administrative work of writing medical certificates for the SIA. Several of the women described experiencing pressure from society as a whole—from the political climate or just strangers in general. They believed these people wanted them to RTW to relieve the financial burden on society. Some women believed their neighbours, who saw the women at home during the day, questioned their sick leave behind their back and in a judgemental way: “I’m afraid people will think, ‘why is she at home now, why isn’t she at work?’ people who see I’m at home during the day. And that they’ll think ‘why can’t she manage this… how difficult can it be’” (14). Several women believed their colleagues wanted them to RTW, mainly because they were appreciated for their special competence and unique personality—or, on the other hand, perhaps just to maintain the staffing. Family and/or close friends were believed to be supporters of RTW because they cared about the women and thought RTW was best for them. However, several women also believed their family and/or close friends wanted them to work to relieve their worries about the sick leave, but also so the women could contribute more to the family economy. Some women believed their partner wanted to have more time alone: “It’s been really hard for him to have me at home. He never gets any peace” (5). Women who worked with clients/service-users/patients believed these people wanted them to work because they appreciated the good work the women do.

#### Non-supporters of RTW

Stakeholders, most often employers, were sometimes also believed to be non-supporters because they thought the women were too sick to work, either temporarily or permanently. Regarding colleagues, the women sometimes believed they were no longer desired at work because of their inability to perform as they had previously: “I know my workmate is having to do a lot of things I can’t manage. I don’t think he wants me to see that it’s difficult” (15). One woman believed that her replacement feared losing her job when the woman returned. Some women believed their children were non-supporters. Not because they did not want their mother to work, but because there were benefiting from having a parent at home all the time: “Well, they (the kids) like it when I’m home when they wake up in the morning and when they come home. They don’t have to go to afterschool activities. My being home is a source of security for them” (5).

### Control Beliefs

Control beliefs concern the facilitators or barriers the women believed affected RTW.

#### Facilitators of RTW

Implementation of individual adaptation regarding rate of increased activity—working tasks or workplace, for example, a “model workplace” specially adapted to people about to RTW—was thought to facilitate RTW: “So it’s a (workplace) for staff with certain problems, and there are trained staff there too who know what they’re doing. So you get out into a regular environment, but you can also slow down a bit” (20). The women believed there were prerequisites for individual adaptation: the employer must know about mental disorders and the women must be allowed to participate in planning their own RTW. Having a rehabilitation plan and regular contact with the employer and stakeholders during sick leave were believed to be necessary facilitators, as was having a well-staffed workplace, because these preconditions allowed the women to have a bad day and leave for a while if needed. The women stated that receiving positive treatment from others was important, such as understanding from family, close friends and stakeholders. They believed it was important for their employer and other stakeholders to be engaged in their situation and to see them as human beings, not only as a case of illness. Furthermore, colleagues’ tolerance of the women’s condition and their longing for the women’s RTW were also thought to be facilitators. Sometimes, conversational therapy and medications could provide necessary relief of the symptoms caused by the CMD. Support from family and close friends enabled the women to rest at home, as someone else could care for the children. The support of one’s partner could also be crucial to getting out of the house and away to work at all:” My husband has driven me to work when things feel tough. He’s made breakfast and done such a lot for me to help me get out the door” (15).

#### Barriers to RTW

Most of the women believed that having high demands was a barrier, such as expectations for rapid RTW or having responsibility for more work tasks than they could manage: “Having only 3 months to get used to—being up to full speed—it’s like from nothing to ‘puff’. It’s horrible, a really big step” (2). Workplace factors, such as understaffing or profit interests on the part of the employer, were believed to contribute to high demands. Having a demanding family life, such as small children at home, was believed to be a barrier, as was financial stress due to low sick-leave benefits or the perceived threat of not receiving any sick leave benefits at all: “You don’t get well by being poor. You don’t get well by having yet another thing to stress out about” (5). Furthermore, the women believed that negative treatment on the part of others was a barrier. It could occur when one’s employer and other stakeholders trivialized the women’s condition and saw them as a scapegoat for financial loss, instead of caring about them as human beings. Several women believed that factors indicating an unhealthy working environment—such as weak leadership, a poor atmosphere among colleagues, bullying and jealousy concerning the women’s workplace adaptations—were also barriers: “They think, well here she comes and gets all these exemptions” (5). Symptoms caused by the CMD, such as tiredness, concentration difficulties and forgetfulness, were also believed to be barriers. Several of the women felt performance anxiety, shame or guilt over their situation and believed this was an overall barrier in life. For example, the women thought others saw CMDs as something “ugly” or that others had preconceived notions about the women taking advantage of the system because they did not want to work. Self-recrimination occurred: “Sometimes I’m ashamed that I couldn’t manage my work. Other people do. Why can’t I?” (14). The women believed their age mattered, especially menopause and being close to retirement, due to their decreased energy or motivation, given that their working life was nearing its end. Younger women, on the other hand, could perceive themselves as too eager to RTW because they were young, or they could feel pressure to live up to their generation’s ideal image: “You’re supposed to work a lot and everything is supposed to be perfect, and then post it all on social media” (4). The women tried to facilitate RTW for themselves using various strategies: accepting one’s limitations, no longer trying to live up to perceived norms in society regarding performance in different areas of life, scheduling gaps in one’s calendar, saying ‘no’ more often, working out and being grateful for every little step forward rather than grumbling over steps not already taken. One woman described how she focused on her strengths instead of distressing over her weaknesses: “Right now I’m not going to improve myself, just do what I’m good at” (18). Some women used their negative experiences as tools to be a more harmonious person or used their increased understanding of mental disorders in their work with people.

## Discussion

The present study aimed to describe, based on the TPB, women’s beliefs about RTW after long-term sick leave due to CMDs. Regarding behavioural beliefs, the women felt that RTW would include them in a meaningful context. This is in line with the well-founded theoretical concept of sense of coherence [25], which emphasizes the importance of coherence and meaning. Furthermore, the women described different sides of the same issue. For example, belonging to a social context and having balance were believed to be advantages, while being with colleagues and difficulties maintaining balance were disadvantages. Concerning normative beliefs, the women believed others’ support regarding their RTW was two-sided, i.e., there was a discrepancy between the women’s and the supporters’ desires, causing the women to feel pressured rather than supported. Regarding control beliefs, the women emphasized high demands and individual adaptation at work. They believed they had their own strategies for facilitating RTW, but having the support of others in overcoming barriers and strengthening facilitators was essential to RTW.

Regarding behavioural beliefs, several women stated that physical activity, socializing and time with one’s children had to receive low priority if they were to have enough energy to RTW. This is in line with previous research showing that women may function very well at work at the expense of lower functioning in private life during RTW [4]. This reveals how important it is for women who RTW to perform well at work. It also shows the importance of adjusting RTW to women’s needs, so that healthy behaviours, as well as family and social life, do not suffer. This may help them achieve a stronger sense of coherence and, therefore, better health [25]. This may be crucial to a sustainable RTW. Furthermore, lack of energy may be due to too early RTW, as also described in a previous study [4]. According to a report from the SIA, individuals whose sick leave application was rejected worked to a lesser extent the following three years than those who completed their sick leave voluntarily [26]. This indicates that sudden and unexpected RTW is more unstable and, hence, that stable RTW requires that more individually tailored support be provided by the employer and the workplace, such as opportunities to rest during workdays.

Although feeling competent was described as an advantage, several women described a fear of loss of competence due to the CMD or their competence not being taken advantage of during RTW. In Sweden today, work tasks during early RTW after long-term sick leave are based on what the individual can do, and these tasks may not necessarily be in his/her field of competence [27]. This suggests that it might be important for adaptation of working tasks to occur within the women’s field of competence. According to the TPB [17], the importance of behavioural beliefs to behaviour is dependent on what values individuals have attached to these beliefs. This may well differ across women. For instance, maintaining one’s competence may be more important for women who work in fields where things develop fast or where routines change frequently, such as nursing or teaching.

Women believed they had supporters as well as non-supporters for their RTW, even though perceived reasons for giving or not giving support varied widely—from being based on love for or appreciation of the women to negative support that made the women feel pressured to act to satisfy others’ needs. This was also described in previous research, where women with a CMD after long-term sick leave felt pressure from the SIA to RTW rapidly, which was experienced as an aggravating demand [13]. To summarize, women in the present study felt pressure to RTW/not RTW from different directions and for different reasons, and when they did RTW, they felt pressure to perform as they had previously. Given that important others’ attitudes towards RTW seem to have a significant impact on individuals’ RTW behaviour [28], the women may find themselves in a very difficult situation, thus revealing the complexity of the normative pressure placed on them. However, we must keep in mind that the present study does not reveal the importance of others’ support (which is required by the TPB as regards determining the level of social pressure). Hence, the impact on RTW based on these beliefs must be interpreted with caution.

Control beliefs showed that having high demands at work was commonly believed to be a barrier and individual adaptation to be a facilitator, which is in line with previous research [15]. Most women in the present study reported having previously been on sick leave owing to a CMD. The occurrence of relapses implies that individual adaptation may be insufficient in some way, i.e., too short or not sufficiently tailored to the women’s needs. Furthermore, an ongoing CMD might in itself impair their ability to adhere to planned facilitating strategies [15]. The results also showed that financial stress was believed to be a barrier, while earlier research has shown that the thought of a higher income after RTW can be experienced as a motivator [14]. Given their symptoms, the threat of withdrawn sickness benefit may be especially stressful for women with CMDs. This highlights the importance of supporting RTW among these women in an individually tailored manner. Surprisingly, though most of the women believed a poor work environment had caused their CMD, some nonetheless thought that changing their thoughts and behaviour in daily life was the solution, which is in line with earlier research [5]. Given this, employers should evaluate the cause of the women’s CMDs and work together with the women to improve this particular area.

### Strengths and Limitations

One strength of the study was its theoretical foundation. The TPB was shown to be a useful theoretical framework for describing women’s beliefs about RTW during or after long-term sick leave due to a CMD. However, by restricting the sample to women, we jeopardize the transferability of the results, as the sick leave population typically consists of both genders. On the other hand, it is valuable to examine the phenomenon in-depth among the most vulnerable group in the context. Because we mixed the beliefs of women currently on sick leave with those of women no longer on sick leave in the analysis process, we do not present which beliefs belong to which group. However, the beliefs were considered to be similar between the groups, which is why we chose to present the results together. Furthermore, looking at a heterogeneous group it is often considered a strength in qualitative research, as it enables researchers to identify common structures across the diversity [23, 29]. Using the TPB in relation to RTW might be complicated, as RTW is not always within the individual’s own control. According to the TPB, actual control over behaviour is essential to performing the behaviour. In other words: If there is no job to return to, RTW is not possible. However, all of the women in the present study were employed, making it relevant to apply the TPB.

### Future Research

Because long-term sick leave for CMDs is increasing among women, there is an urgent need to conduct further research in this area. The present study provides a starting point for future quantitative research, which could focus on identifying important TPB predictors of RTW or on intervention studies. Furthermore, the present study offers a picture of which beliefs are important for stakeholders to target when working with RTW promotion among these women. The contradictory support and control beliefs should be given particular attention. Moreover, research evaluating RTW stakeholders’ opportunities and barriers regarding meeting these women's needs is needed.

## Conclusion

Starting from the TPB, the present study contributes new findings on women’s beliefs about RTW after long-term sick leave for CMDs. The women were shown to value their work highly. They tried to find ways to return sustainably by balancing the demands they believed others had with their own abilities, regarding RTW or performance at work. Based on these findings, we suggest that various RTW stakeholders should strive to provide the tasks and work pace the women need, allowing the women to maintain their professional competence and a sense of meaning.

## Data Availability

The datasets generated during and/or analysed during the current study are available from the corresponding author on reasonable request.
